# Extracurricular Psychiatry Enrichment Programmes for Undergraduate Medical Students: A Systematic Review

**DOI:** 10.1192/bjo.2026.11153

**Published:** 2026-06-30

**Authors:** Sharna Bennett, Tiago Gameiro-Inacio, Joanne Rodda

**Affiliations:** 1 Kent and Medway Mental Health NHS Trust, Kent, United Kingdom; 2 Kent and Medway Medical School, Canterbury, United Kingdom

## Abstract

**Aims:**

Psychiatry is a rewarding career which has grown in popularity in recent years. Despite psychiatry training posts recently achieving high fill rates, the specialty has consistently faced long-standing recruitment difficulties, with associated negative perceptions and low interest from medical students. Exposure to extracurricular enrichment programmes has been suggested as a potential method to improve student attitudes, increase engagement, and encourage early career interest in psychiatry. Extracurricular enrichment programmes offer medical students the opportunity to engage with psychiatry beyond the mandatory curriculum, such as through summer schools, special study modules and mentoring schemes. This systematic review aims to evaluate the current literature on extracurricular psychiatry enrichment programmes for undergraduate medical students.

**Methods:**

The review was registered with PROSPERO (CRD420251025990) and followed PRISMA guidelines. Four databases were searched (Embase, Ovid MEDLINE, APA PsycInfo and ERIC) using pre-determined search strings. Records were screened and data extracted independently by two authors. Extracted data included programme characteristics, student experiences, attitudes towards psychiatry and mental illness, career intentions and eventual specialty outcome. Methodological quality was appraised using the Mixed Methods Appraisal Tool (MMAT). Due to heterogeneity in study designs and outcomes, findings were synthesised narratively to identify patterns and themes.

**Results:**

Of 731 articles screened, 65 full reports were assessed for eligibility and 27 were included. These studies were conducted across 10 countries, most commonly the USA and UK. Programme duration and format varied from single-day events (n=2) to longitudinal mentoring schemes spanning medical school (n=5). Summer schools were most common (n=10). Other schemes included mentoring, elective courses, special study modules, student-led clinics and film/media-based activities. Student feedback was positive, particularly for mentoring and film/media initiatives. Most studies reported improved attitudes towards psychiatry and mental illness, with several reporting statistically significant improvements using validated tools. Of 18 studies assessing career intentions, 16 reported increased interest in a career in psychiatry, and six studies reported higher subsequent match rates into psychiatry specialty training.

**Conclusion:**

Although potential self-selection bias may arise from programmes that are both voluntary and extracurricular in nature, the available literature suggests that these programmes provide positive student experiences, and foster improved attitudes and early interest in psychiatry through meaningful learning opportunities. However, longitudinal research is needed to clarify the long-term impact, particularly on those without prior interest. Despite study heterogeneity, this review highlights the importance of enrichment programmes to support recruitment, provided inclusivity and accessibility are considered during programme development.

